# Analysis of starch grains trapped in human dental calculus in Áspero, Peru during the Initial Formative Period (3000–1800 BCE)

**DOI:** 10.1038/s41598-023-41015-6

**Published:** 2023-08-29

**Authors:** Marco Yseki, Luis Pezo-Lanfranco, Marco Machacuay, Pedro Novoa, Ruth Shady

**Affiliations:** 1 Zona Arqueológica Caral, Unidad Ejecutora 003, Ministerio de Cultura del Perú, Lima, Peru; 2 Institute of Environmental Science and Technology (ICTA), Universitat Autònoma de Barcelona, Barcelona, Spain; 3 Escuela Profesional de Arqueología, Facultad de Ciencias Sociales, Universidad Nacional Mayor de San Marcos, Lima, Peru

**Keywords:** Archaeology, Plant domestication

## Abstract

The objective of this research is to identify the plants consumed and to determine their dietary importance in Áspero, an urban center on the coast of the Supe Valley, Peru. Consequently, starch grains trapped in the human dental calculus of nine individuals were recovered, while the results from one individual from the Sacred City of Caral, located in the interior of Supe Valley, are presented. Eight species of food plants were identified, among them C_3_ plants: sweet potato, squash, potato, chili pepper, algarrobo, manioc and bean and C_4_ plant: maize. Previous isotopic analysis indicates that C_3_ plants formed the foundation of the diet at Áspero and Caral. Our results indicate a high ubiquity of C_3_ plants like sweet potato (100%) and squash (90%) suggesting, with caution, that these taxa were an important C_3_ source in the menu. Maize, C_4_ plant, showed a similar ubiquity (100%) to sweet potato and squash, however, previous isotopic analysis indicate that maize was a marginal food in Áspero and Caral. These results support that the absence and abundance of starch grains cannot be employed to directly infer the frequency of intake of C_3_ and C_4_ plants within a small population, as suggested by previous studies.

## Introduction

**Figure 1:**
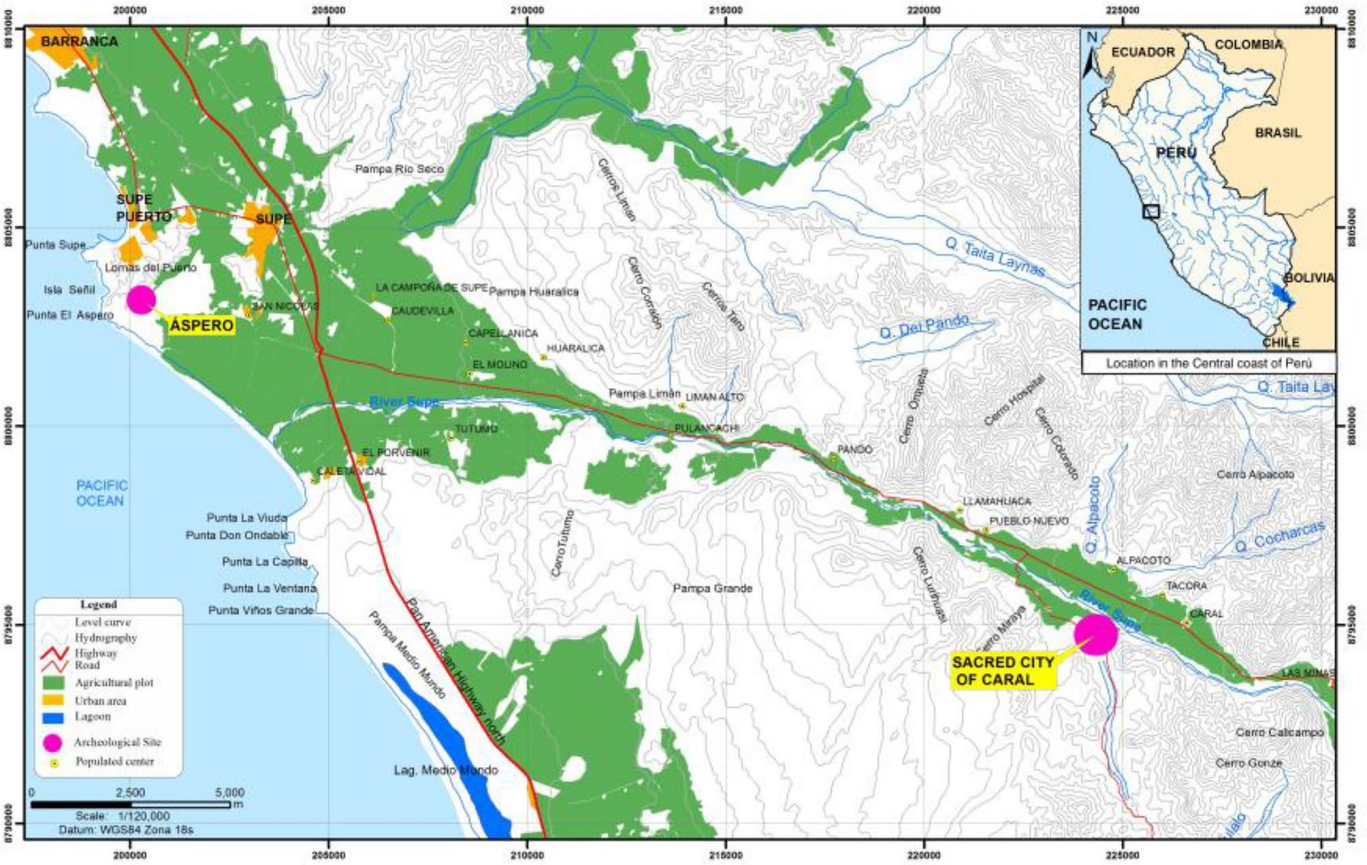
Location of Áspero and Sacred City of Caral in the Supe Valley, Peruvian Central Coast. The figure was generated by geospatial analysis of ArcGIS software (version ArcGIS 10.3; http://www.esri.com/software/arcgis/arcgis-for-desktop) and the map data was obtained from the IGN (https://www.idep.gob.pe/geovisor/VisorDeMapas/) and MTC (https://portal.mtc.gob.pe/estadisticas/descarga.html) portal.

Extensive archaeological research conducted over the past three decades in the Central Andes has profoundly changed our understanding of the process of plant domestication, the adoption of farming, the nuances of changes in subsistence strategies over time and their relationship to processes of social complexity^1–8^. Current data suggest that during the Initial Formative period (3000–1800 BCE), the Supe Valley of the north-central Peruvian coast witnessed the rise of the Sacred City of Caral and 24 other associated urban centers with monumental architecture, which constitute the tangible evidence of Caral, the earliest civilization of the Americas^5,9^. Among these archaeological sites, two of the utmost importance in the discussion of the origin of early civilization are Áspero and Sacred City of Caral. Áspero, a coastal settlement (Fig. 1) generally dated to ~ 3000 BCE, has been considered for the last 50 years the archetypal site for the *Maritime Foundations of Andean Civilization* hypothesis (MFAC)^10,11^, which argues that the earliest complex societies were initially based on the exploitation rich shoals of little fishes and other endemic marine species, adopting later agriculture to maintain the social structures previously institutionalized. Since Áspero yielded the earliest radiocarbon dates for any site on the coast and the Supe Valley, it was considered as the locality in which the first complex society possibly emerged in the region^12,13^. However, new research has shown that, although the human occupation of Áspero started earlier, the construction of monumental buildings at the site is later than at Sacred City of Caral^14^. Sacred City of Caral is the main settlement of the middle Supe Valley (Fig. 1) and shows clear evidence of early urbanization and the largest amount of labor invested among all the sites of the valley. Thanks to more than 25 years of continuous archaeological research with excavations, Caral is currently recognized as the main site of the Supe Valley and possibly the capital of a regional polity between 3000 and 1800 BCE. The data from Caral changed the dominant conceptions on the regional process, challenging the plausibility of the MFAC and re-fueling the debate on the geographic origin (if coastal or inland?), the economic basis (farming-based or fishing-based?), and political nature (heterarchy, chiefdoms, pristine state?) of the earliest Andean complex societies^8,15–18^.

The macrobotanical remains recovered in Áspero and Sacred City of Caral, suggest early cultivation and consumption practices of several plant species in Supe Valley^4,5,12,14,19^. In qualitative terms, the plant inventories of Áspero and Sacred City of Caral are similar. In Áspero and Caral, the following food plant remains were recovered: guava (*Psidium guajava*), squash (*Cucurbita* sp.), *achira* (*Canna indica*), sweet potato (*Ipomoea batatas*), potato (*Solanum tuberosum*), *oca* (*Oxalis tuberosa*), bean (*Phaseolus vulgaris*), lima bean (*Phaseolus lunatus*), chili pepper (*Capsicum* sp.), *pacay* (*Inga feuillei*), *lucuma* (*Pouteria lucuma*), avocado (*Persea americana*), *guanábana* (*Annona muricata*) and maize (*Zea mays*)^4,5,18,20,21^. A recent study, based on isotopic analysis, suggests a high intake of C_3_ carbohydrates (tubers, legume, cucurbit and fruits) at Áspero and Sacred City of Caral^18^. However, microbotanical analysis is required to identify which C_3_ plants were consumed and if there was predominance of some species, that in turn, can lead us to better understand of their economic relevance and correlated effects in land use, labor organization and trade networks. In the last decades, starch grains from dental calculus have been used as a dietary proxy of archaeological interest. The human dental calculus offers an excellent opportunity to better comprehend plant consumption, food processing and cooking techniques^22–27^. In this study, we present the results of the first analysis of starch grains in human dental calculus from the Áspero site. This analysis aims to identify which plants were consumed by the coastal populations that Supe Valley during the Initial Formative Period, to complement the results of previously published macrobotanical analysis and based on the ubiquity (%) of the C_3_ taxa identified, to determine the C_3_ plants that constituted the main vegetal source of diet at Áspero and Sacred City of Caral, as suggested by isotopic analyses^18^.

## Results

Starch grains were recovered from all teeth analyzed. A total of eight taxa of edible plants were identified, including sweet potato (*Ipomoea batatas*), squash (*Cucurbita* sp.), potato (*Solanum tuberosum*), chili pepper (*Capsicum* sp.), algarrobo (*Prosopis* sp.), manioc (*Manihot esculenta*), bean (*Phaseolus* sp.), maize (*Zea mays*) and specimens of the Fabaceae family (Table 1).

**Table 1:** Identified taxa per burial and the number of individual starches observed.

Site	Áspero	Áspero	Áspero	Áspero	Áspero	Áspero	Áspero	Caral	Áspero	Áspero	Total	%	Ubiquity (%)
Chronology	3000–2700 BCE	2907–2575 cal BCE	2700–2200 BCE	2669–2306 cal BCE	2400–2200 BCE	2700–2100 BCE	2700–2100 BCE	2398–2038 cal BCE	2200–1900 BCE	2200–1900 BCE			
Burial	ZAC 1710	ZAC 3024	PEACS 4439	ZAC 6360	ZAC 2996	ZAC 1693	ZAC 3021	CAR 391	PEACS 2140A	PEACS 2960A			
*Capsicum sp.*		3		1	2	2					8	3.6	40
*Cucurbita sp.*	3	12		6	2	6	2	12	7	4	54	24.4	90
*Fabaceae*	1			1			1			1	4	1.8	40
*Ipomoea batatas*	2	8	2	7	2	10	3	6	23	5	68	30.8	100
*Manihot esculenta*								1			1	0.5	10
*Phaseolus sp.*			1	6							7	3.2	20
*Prosopis sp.*	1			1			2		1		5	2.3	40
*Solanum tuberosum*	1	1		3		1		1			7	3.2	50
*Zea mays*	2	5	7	1	1	4	3	5	6	2	36	16.3	100
Unidentified	2	2	2	5	3	5	1	6	3	2	31	14.0	
Total	12	31	12	31	10	28	12	31	40	14	221	100	

At least 221 starch grains were recovered, identified and recorded (Table 1). Sweet potato (30.8%), squash (24.4%) starch grains were the most common in human dental calculus. We recovered starch grains that could not be identified (14.0%), due to the absence of diagnostic features or because they are not represented in our current reference collection or in other published works. Table 1 shows the ubiquity of the food taxa identified at Áspero and Caral, Supe Valley. Sweet potato (100%), maize (100%) and squash (90%) showed high ubiquity during the Initial Formative Period (3000–1800 BCE).

**Figure 2:**
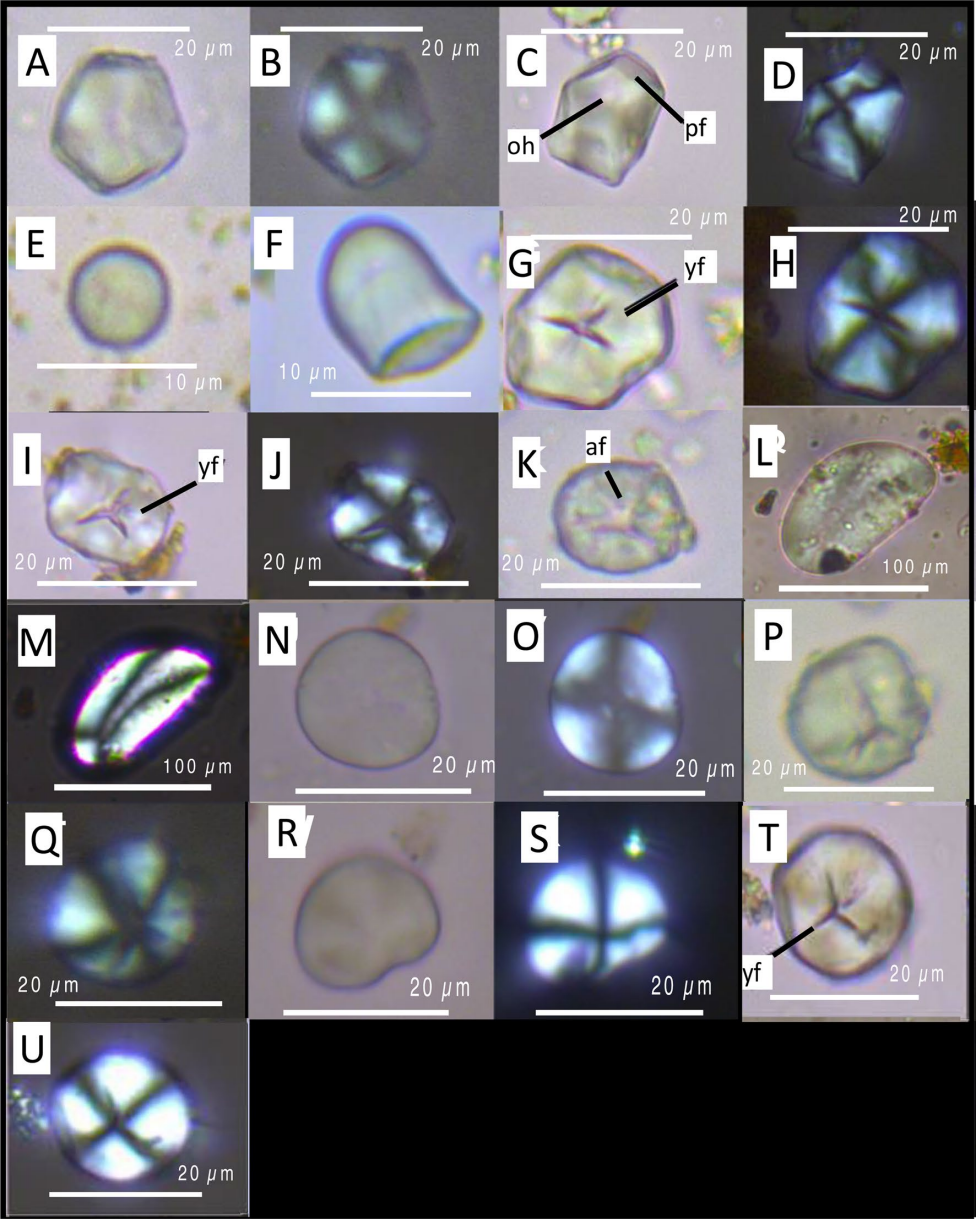
Sweet potato starch grains (**a**–**d**) with diagnostic polyhedral shapes with pressure facets (pf) and open hilum (oh). Squash starch grains (**e**,**f**), starches are spherical and bell-shaped with a cap-like distal end and eccentric hilum. Maize starch grains (**g**–**k**), starches are polygonal (**g**–**j**) and oval shapes (**k**) with “y” fissures (**g**,**i**,**l**). Maize starch grain showing furrowing damage possibly from fermentation (**k**). Potato starch grains (**l**,**m**). Chili pepper starch grains (**n**,**o**). Algarrobo starch grains (**p**,**q**). *Phaseolus* sp. starch grains (**r**,**s**). Manioc starch grain with diagnostic bell-shaped showing “y” fissure (**t**,**u**).

Sweet potato starch grains presented a mean size of 19.8 ± 6.7 μm and have polygonal shapes with open hilum in many cases. They showed two or three pressure facets (Fig. 2a–d). The mean size of squash starch grain was 8.3 ± 1.9 μm. Squash starch grains are spherical and bell-shaped with a cap-like distal end and eccentric hilum (Fig. 2e–f).

Maize starch grains are polygonal, spherical, and oval shapes with different fissure variants (transverse, asymmetric, X-shape and Y-shape). The mean size of maize starch grain was 18.2 ± 6.3 μm (Fig. 2g–k). Potato starch grains presented a mean size of 93 ± 4.3 μm and have oval shapes with eccentric position of the hilum and lamellae visible (Fig. 2l–m). Chili peppers starch grains are lenticular shaped with a central depression. The mean size of chili peppers starch grain was 18.2 ± 2.1 μm (Fig. 2n–o). Algarrobo starch grains presented a mean size of 14.4 ± 3.9 μm and ovoid and irregular (with facets irregularly disposed) shapes with different fissure variants (transverse and star-shape). Algarrobo starch grains present a high birefringence and broken Maltese cross in grains with irregular forms under polarized light (Fig. 2p,q). Bean starch grains are oval shaped, mostly kidney-shaped with a mean size of 19.7 ± 2–4 μm (Fig. 2r,s). Some bean starches presented a linear fissure. The one manioc starch grain recovered is bell-shaped (18.1 μm) with Y-shaped fissure (Fig. 2t,u).

## Discussion

To start with our discussion of the findings we need to consider some limitations of the method. The pathways of inclusion of starch granules of different species in the dental calculus matrix are different and a specific “rate” has never been calculated. Theoretically, this property depends on biological individual factors (saliva, dental anatomy, microflora) and several physical attributes of food related to cooking methods (texture, if raw or gelatinized), sugar and fiber content of the species, etc. and needs more experimental work to be elucidated^28^. Thus, the presence of starch of edible plants trapped in dental calculus only provides confirmation that some specific species were introduced in the mouth, chewed, and possibly ingested. We cannot know if they represent more or less consumption in absolute quantitative terms. To unravel this, other sources of evidence are necessary. In addition, previous studies report that the production and the density of starch grain in specialized storage organs are variable among plant families and even among species of the same genus^29^. Therefore, it is not feasible to estimate the contribution of a specific starchy plant to the diet of an individual or population using starch numbers recovered from different taxa. To evaluate the relative significance of certain starchy plants in the diet, previous studies^24,30,31^ have used the ubiquity value of each taxon in the set of samples analyzed, assuming that each sample represents an individual and the more ubiquitous, the more frequently it was probably to be used or consumed. In this section we will discuss with caution, based on the ubiquity (%) of the taxa identified in the set of individuals from Áspero during the Initial Formative Period, the contribution of these plants in the vegetal component of the diet of the inhabitants of Supe Valley.

Various plants identified in the human dental calculus are consistent with the macrobotanical evidence reported in Áspero and Sacred City of Caral sites^4–6,20^. Nonetheless, there are some taxa identified in this study that were not reported in the macrobotanical record. For example, manioc remains were not recovered and sweet potato and potato macroremains are relatively scarce in Supe Valley^4–6,20^. The absence or low number of recovered tubers among the macroremains can be associated with poor preservation and/or total consumption. On the other hand, guava starches were not reported in our analysis, however, remains of seeds of this fruit are very abundant in the macrobotanical record from Áspero and Sacred City of Caral^4–6,20^. This discrepancy probably could be explained by issues of starch preservation in the matrix of the dental calculus, a topic still poorly explored. These results highlight the importance of performing several complementary analyses.

Our microbotanical analysis indicate a high ubiquity of C_3_ plants like sweet potato (100%) and squash (90%) (Table 1). Likewise, previous isotopic analyses indicate that C_3_ plants formed the basis of the diet at Áspero and Sacred City of Caral^18^. The most important contribution of this study is the identification of the specific taxa of C_3_ plants consumed (sweet potato, squash, potato, chili peppers, algarrobo, beans and manioc). Based on ubiquity, our findings, with caution, suggest that the C_3_ tuber more conspicuous in Áspero was sweet potato, and that squash was also an important C_3_ source in the menu. Other C_3_ tubers identified were potato and manioc and showed a moderate (50%) and low ubiquity (10%), respectively (Table 1). The low ubiquity of manioc throughout the Initial Formative Period in the Supe Valley could possibly be interpreted as a more occasional consumption. Because the center of domestication of manioc is considered the southern amazon^32,33^ and manioc showed a low ubiquity at Supe Valley, it is likely that the inhabitants of the Supe Valley acquired manioc through exchange from other regions. Potato is believed to have been domesticated in southern-central Andes^34^. Despite their original center of domestication, potato, as today, can grow in middle coastal valleys. Since potato showed a moderate ubiquity, it is plausible that inhabitants of Áspero acquired potato through exchange from middle coastal valleys from Supe Valley.

Other common C_3_ species of Initial Formative Period identified in this study, also reported in macrobotanical analyses in Áspero and Sacred City of Caral^4–6,20^, are chili peppers and legumes. Chili peppers presented moderate ubiquity (40%), while bean showed low ubiquity (20%). In contrast, a previous study of plant micro-remains trapped into dental calculus indicates that legumes and squashes were the major dietary sources in northern Peru (Nanchoc Valley, 6210–4970 BCE)^23^. During the Initial Formative Period, at least in the coastal of Supe Valley, a more varied starchy food diet based mainly on sweet potato and squash is noticed, suggesting a temporal trend of change in starchy food diet over the time. It is possible that changes in the subsistence economy with a growing dependence on crops during the Initial Formative Period (farming intensification), was the cause of the differences in sources of starchy food diet between the inhabitants of the Supe Valley and other earlier archeological sites.

Remains of maize cobs were recovered in Áspero 80 years ago^35^. However, the archeological context of these findings is uncertain and was the motif of heated debates^36–38^. Our results indicate that maize starch grains were recovered from burials dated to early stages of the Initial Formative Period (3000–2700 BCE) in Áspero. In Caral, based on macro-botanical evidence, Shady^21^ suggests that maize was incorporated from 2300 BCE. The few remains of maize cobs recovered in Caral are varied, but when compared to other food plants suggest that was a less important component of the population's diet^5,21^. In addition, the association of maize with ritual contexts was recurrent in Caral^21^. The maize starch grains recovered in this study are related to the Middle Expansive and Late Caral periods (2398–2038 cal. BCE) (Table 1). Our results indicate a consumption of maize at Supe Valley during the Initial Formative Period and maize showed a high ubiquity (100%), similar to C_3_ plants (sweet potato and squash) (Table 1). However, previous isotopic analyses suggest that maize was a marginal food (< 12% of calories) in the Supe Valley during the Formative Period^18^. Our results and previous studies indicate that the absence or abundance of starch grains cannot be used to directly infer the frequency of C_3_ and C_4_ plant consumption in a small population^39–41^. We recovered a one starch grain with damage by a possible fermentation process at Sacred City of Caral. The CAR 391 individual was directly dated 2398–2038 cal. BCE and was recovered from sacrificial contexts^42^. The starch presented radial striations (furrowing lines) and an irregular extinction cross, similar to starch grains with evidence of fermentation damage reported during the Inca period^43,44^. However, the type of damage in maize starch grain in CAR 391 burial has also been observed during maize milling process^30^, although, another study suggests differences in the damage produced in maize starch by fermentation and milling^44^. Our results cannot affirm that maize was fermented for the preparation of *chicha* (maize beer) in Caral during the Initial Formative Period. In order to confirm that the inhabitants of the Supe Valley fermented maize it is necessary to perform future analyses of microremains and micromolecules in an expanded sampling in the Supe Valley that includes individuals and storage of maize beer (e.g., gourds). In addition, further experimental works using different maize varieties are needed to explore the different kinds of possible damage to maize starch grains.

## Conclusions

The large number of plants identified shows that the inhabitants of the coastal Supe Valley consumed a variety of starchy plant foods. Our results indicate that the inhabitants of Áspero consumed C_3_ (sweet potato, squash, potato, chili peppers, algarrobo, beans and manioc) and C_4_ (maize) plants. Previous isotopic analyses indicate that C_3_ plants formed the basis of the diet at Áspero and Sacred City of Caral^18^. Our findings show that sweet potato and squash were highly ubiquitous in the set of individuals analyzed at Áspero suggesting, with caution, that these C_3_ taxa plants may have contributed more to the total vegetable component of the diet as a group. However, maize presented a high ubiquity similar to sweet potato and squash, although previous isotopic analyses indicate that maize was a marginal food in the Supe Valley^18^. These results support the idea that the analysis of starch alone should not be used to determine the contribution of C_3_ and C_4_ plants in the vegetable diet, especially when sample sizes are small. Starch analysis offers opportunities to identify some of the starchy plants consumed, while stable isotope analysis provides insights into the relative proportions of different food sources in an individual's diet, being necessary to perform these complementary analyses to better understand the diet of ancient peoples.

## Material and methods

### Study area

Áspero is located 5 km north of the Supe River drainage, on the southeastern slope of a natural elevation, just over 500 m from the ancient beach (Feldman, 1980; Shady and Cáceda, 2008), at WGS84 coordinates 77°44′31″ W and 10°48′52″ S, and 30 masl (Fig. 1). During the Initial Formative Period, Áspero was associated with the sociopolitical system of Caral^20^, being an urban center with 30 architectural assemblages dispersed in 18.75 ha, including four stepped pyramidal buildings, residential sectors, warehouses, a central space with public buildings and two sunken circular plazas^20^.

Sacred City of Caral is situated 26 km inland from the Pacific coast over an alluvial elevated terrace of the left margin of the river, in the middle section of the Supe Valley^4,5^, in coordinates WGS84 77°31′20″ W and 10°53′30″ S, at 360 masl (Fig. 1). Caral is a planned urban center with 35 architectural complexes that contains a central zone of monumental architecture with four main pyramids, two circular sunken courts, a big square and several administrative buildings, as well as areas with residential and non-residential architecture^4,5^. Radiocarbon dates from Caral indicate that it was occupied during the Formative Initial Period (2860–1970 cal. BCE)^4^. In the Supe Valley, Caral was the center of the greatest economic, social, political, and religious dynamism during the third millennium BCE^5^.

### Burial contexts

Human dental calculus from ten burial contexts (Table 2) excavated by Shady and colleagues from Áspero (9) and Sacred City of Caral (1) were selected considering the following inclusion criteria: (a) teeth with visible and well-preserved dental calculus; (b) ^14^C AMS and/or relative dating by association; (c) isotopic and other bioarcheological data, if available. The funerary contexts analyzed in this work were previously classified to the Initial Formative Period^18^. Dental material and bone from three burial contexts (ZAC 3024, ZAC 6360 and CAR 391) provided only three direct AMS dates calibrated, while the remaining seven burial contexts were chronologically classified based on contextual associations through several radiocarbon dates obtained by the Caral Project during the last 25 years^18^. Due to the difficulty of recovering human remains from the Initial Formative Period, the analysis of dental calculus from burial contexts of Áspero and Sacred City of Caral offers a unique opportunity to know what plants were consumed by the populations of these in political systems of rising complexity.

**Table 2:** Individuals analyzed. The chronology of ZAC 3024, ZAC 6360 and CAR 391 individuals was obtained from direct dating of the burial bones.

Nº	Site	Burial	Sex	Chronology
1	Áspero	ZAC 1710	Female	3000–2700 BC
2	Áspero	ZAC 3024	Female	2907–2575 cal. BC
3	Áspero	PEACS 4439	Female	2700–2200 BC
4	Áspero	ZAC 6360	Male	2669–2306 cal. BC
5	Áspero	ZAC 2996	Unknown	2400–2200 BC
6	Áspero	ZAC 1693	Child	2700–2100 BC
7	Áspero	ZAC 3021	Female	2700–2100 BC
8	Caral	CAR 391	Male	2398–2038 cal. BC
9	Áspero	PEACS 2140	Child	2200–1900 BC
10	Áspero	PEACS 2960	Child	2200–1900 BC

The chronology of the other individuals was obtained by contextual association.

*Source* Pezo-Lanfranco et al.^18^, Supporting Information S1c.

According to Directorial Resolution 202–2020 of the Peruvian Ministry of Culture, the Caral Archaeological Zone is authorized to conduct archaeological excavations in the Supe Valley. All methods were carried out in accordance with relevant guidelines and regulations of Caral Archaeological Zone and Peruvian Ministry of Culture.

### Sample extraction and starch grain recovery

A total of twenty teeth (two per burial context) were analyzed. Individuals were carefully excavated using clean tools and nitrile gloves. The different components of the individuals were wrapped in aluminum foil and plastic bags to avoid contamination and sent to the laboratory of the Caral Archaeological Zone. Considering the intrinsic value of the samples, we used a conservative and non-destructive method to extract calculus and isolate starch grains^22,23^. This method ensures that the starch grains will not suffer damage. First, we used a soft toothbrush and distilled water to remove particles and adherent soil. Then, we used a dental pick to scrape the areas of the teeth with visible calculus, and the residue was transferred directly to a microscope slide on which a few drops of water had been placed. This procedure was performed repeatedly until all the visible calculus was removed. Before the coverslip was put on, one drop of 50% water/glycerin was added to the residue-water suspension. The sampling ended when no more visible calculus was observed. We used sterile and news tools and nitrile gloves during the sampling and slide mounting. All methods were carried out in accordance with relevant guidelines and regulations of Caral Archaeological Zone and Peruvian Ministry of Culture.

Following Diehl^45^ the ubiquity (%) of the plants identified in the set of individuals analyzed was calculated using the formula:

$$U \; taxon=\frac{N \; taxon}{N \; total}\times 100,$$

where *U*_taxon_ is the ubiquity of the plant, *N*_taxon_ the number of burials in which it is found and *N*_total_ is the total number of burials analyzed. The ubiquity measurement identifies how commonly a particular taxon is represented in the set of samples analyzed.

### Taxonomic identification of the recovered starch grains

The morphometric features recorded were size, shape, border, facets, lamellae, fissure, hilum type, and extinction cross arms morphology. To identify recovered starch grains, we used a modern reference collection from Palynology and Paleobotany Laboratory, (LPP-UPCH) and published sources^46–54^. When a diagnostic feature was absent, category unidentified was used. The taxonomic identification of the starch grains was carried out by using a 200–400 × compound microscope under both standard and polarized light (LEICA model DM750P). The analysis was carried out in the laboratory of the Caral Archaeological Zone. The taxa identified in this study form part of LPP reference collection and published sources.

## Data Availability

All data that support the findings of this study within the manuscript and the analyzed individuals are housed in the laboratories of Caral Zone Archaeological, Lima, Peru. https://www.zonacaral.gob.pe/.
